# Designing and Testing Broadly-Protective Filoviral Vaccines Optimized for Cytotoxic T-Lymphocyte Epitope Coverage

**DOI:** 10.1371/journal.pone.0044769

**Published:** 2012-10-03

**Authors:** Paul W. Fenimore, Majidat A. Muhammad, William M. Fischer, Brian T. Foley, Russell R. Bakken, James R. Thurmond, Karina Yusim, Hyejin Yoon, Michael Parker, Mary Kate Hart, John M. Dye, Bette Korber, Carla Kuiken

**Affiliations:** 1 Theoretical Biology and Biophysics Group, Los Alamos National Laboratory, Los Alamos, New Mexico, United States of America; 2 Virology Division, United States Army Medical Research Institute of Infectious Diseases, Fredrick, Maryland, United States of America; 3 Fly Base, Indiana University, Bloomington, Indiana, United States of America; 4 DynPort Vaccine Company, Frederick, Maryland, United States of America; Fudan University, China

## Abstract

We report the rational design and *in vivo* testing of mosaic proteins for a polyvalent pan-filoviral vaccine using a computational strategy designed for the Human Immunodeficiency Virus type 1 (HIV-1) but also appropriate for Hepatitis C virus (HCV) and potentially other diverse viruses. Mosaics are sets of artificial recombinant proteins that are based on natural proteins. The recombinants are computationally selected using a genetic algorithm to optimize the coverage of potential cytotoxic T lymphocyte (CTL) epitopes. Because evolutionary history differs markedly between HIV-1 and filoviruses, we devised an adapted computational technique that is effective for sparsely sampled taxa; our first significant result is that the mosaic technique is effective in creating high-quality mosaic filovirus proteins. The resulting coverage of potential epitopes across filovirus species is superior to coverage by any natural variants, including current vaccine strains with demonstrated cross-reactivity. The mosaic cocktails are also robust: mosaics substantially outperformed natural strains when computationally tested against poorly sampled species and more variable genes. Furthermore, in a computational comparison of cross-reactive potential a design constructed prior to the Bundibugyo outbreak performed nearly as well against all species as an updated design that included Bundibugyo. These points suggest that the mosaic designs would be more resilient than natural-variant vaccines against future Ebola outbreaks dominated by novel viral variants. We demonstrate *in vivo* immunogenicity and protection against a heterologous challenge in a mouse model. This design work delineates the likely requirements and limitations on broadly-protective filoviral CTL vaccines.

## Introduction

The filoviruses (ebolaviruses and marburgviruses) have caused lethal human outbreaks since 1967 [1], devastating primate epizootics in Africa [2], as well as several non-human primate outbreaks originating in the Philippines [3]; they have also been implicated in a livestock epizootic in the Philippines [4]. Filovirus disease in primates is generally a severe hemorrhagic fever syndrome that has no proven specific treatments [5]. The total number of human cases of filoviral disease is near 2,500, spread over roughly 30 outbreaks and laboratory accidents [3]. Filovirus disease is often lethal; the human fatality rate averaged over all known cases is in the vicinity of 70% [3]. Because of the recurrently emergent and extremely serious nature of this disease, significant effort has been made to develop vaccines. However, a pan-filoviral vaccine, or even a clear characterization of what would be required to make one, remains elusive. Here we report theoretical vaccine designs using mosaic techniques first applied to the hyper-variable human immunodeficiency virus type 1 (HIV-1) [6] and preliminary experimental results. While the techniques used here are very similar to those used for HIV-1 mosaic vaccine design, a pattern of repeated introductions of the filoviruses into humans (and primates generally) gives a crucial difference from HIV-1. HIV-1 shows great diversity within the pandemic, but that diversity has developed continuously, leaving intermediate isolates in its wake. In contrast, known filovirus diversity has episodically increased as new outbreaks are found to result from novel viruses, lacking intermediates. This crucial difference is reflected in the phylogeny of the viruses, discussed below. Using the mosaic technique we give the first characterization of the likely requirements for a broadly-protective filoviral vaccine; we also discuss potential limitations on pan-filoviral vaccines and report *in vivo* murine antigenicity and protection.

In the family *Filoviridae* there are currently six species recognized as causing disease in primates: *Marburg marburgvirus, Zaire ebolavirus, Sudan ebolavirus, Reston ebolavirus, Taï Forest ebolavirus* (formerly *Cote d'Ivoire ebolavirus*), and *Bundibugyo ebolavirus* [7]. The genetic diversity of these viruses is large. Protein sequence phylogeny provides a measure of the diversity of protein sequences that is relevant to designing recombinant mosaic proteins. Phylogenetic trees based on protein sequence alignments of filoviral glycoprotein (GP) and nucleoprotein (NP) and the equivalent HIV-1 M-group proteins Env and Gag (current mosaic vaccine targets) show that the diversity of proteins within the Ebolavirus genus is comparable to the protein diversity within the HIV-1 M-group (Figure S1). Including *Marburgvirus* sequences increases the protein phylogenetic diversity beyond what is observed in HIV-1 M-group proteins. The relatively sparse and long branches in the filovirus trees as compared with the HIV-1 trees are a marked difference in their phylogeny. While one can choose a vaccine target population with diversity comparable to HIV-1 (e.g. *Ebolavirus*), there may be additional challenges arising from the notable differences in their phylogeny.

The human fatality rate after infection with the members of the five non-Reston filoviral species appears to be outbreak dependent, and in large outbreaks (>10 cases) ranges from 20–90%. Reston virus has not caused recognized human clinical cases, although there is serological evidence for human infection (i.e. subclinical infection). The human fatality rate from Taï Forest virus disease is unclear, as only a single human case is known. The remaining viruses (Zaire, Sudan, and Bundibugyo viruses, and Marburg virus) give human fatality rates ranging from 34% to 82%, averaged over all known cases [3]. Fatality rates in wild non-human primates are not well documented, but epizootics are thought to be responsible for precipitate declines in primate populations in Africa [2]. Even though fatality rates in large outbreaks of this serious disease are high by any reasonable measure, the strong variation in fatality rates between viral species and between outbreaks, indicates the importance of genetic variation for the course of the disease.

In the context of providing broad vaccine coverage, it is worth emphasizing the apparently idiosyncratic nature of the filoviruses. In recent decades, previously-known filoviruses have continued to re-emerge, sometimes in unexpected ways, as with “pig Ebola” [4] in the Philippines, and new strains have also emerged [8], [9], including a virus in a new genus and species, *Lloviu cuevavirus* [10]. This new virus, identified in European bats, is as phylogenetically distant from marburgviruses and ebolaviruses as they are from each other. The first filovirus discovered, Marburg virus, is still a phylogenetic outlier, even after the discovery of half a dozen other filovirus varieties. While a major reservoir in Africa is likely to be closely connected to African bats [11], [12], known bat strains have not reproduced the full diversity of known filovirus strains, much less anticipated new ones (e.g. Bundibugyo virus). On the other hand, for the most geographically distant filovirus (Reston virus, traced to the Philippines), no natural reservoir has been found, and no plausible explanation has been given to tie Reston virus to a hypothetical origin in Africa, even though it is a member of the genus *Ebolavirus*, an otherwise African taxonomic grouping. Clearly, the filovirus reservoir and the sequence diversity of the viruses are not fully understood. The implication is that a vaccine against the filoviruses should strive for good coverage of common epitopes from the maximum number of types and strains currently available, in the hope that future outbreaks will retain these elements, so the vaccine will still be effective when challenged by a novel strain in a new outbreak. However, the natural history of repeated filovirus introductions to primate populations leads to marked differences in the phylogeny in the form of long sparse branches (see Figure S1), and is likely to have numerous implications for vaccines.

The large genetic variation between known filovirus species, the recurrent emergence of viruses from different species, and the failure to date to produce a filovirus vaccine that protects across all six species [13] indicates that new strategies for vaccine development should be considered. One such strategy was developed by our group for HIV-1 [6], [14], [15], [16]. By maximizing the vaccine's coverage of common potential T cell epitopes found throughout the viral population, the mosaic vaccine strategy is intended to optimize T-cell immunity. In recent years T-cell immunity has been found to be important and effective in combating many viral infections, including filovirus infections. Immune responses to vaccines can confer protection from the lethal effects of ebolaviruses in animal models [17], and cytotoxic T lymphocyte (CTL) responses have been specifically shown to confer protection [18], [19]. Furthermore, inter-strain CTL-mediated protection in non-human primates has also been demonstrated using nucleoprotein (NP) and glycoprotein (GP) as antigens [20]. Promising cross-protection results for a recombinant adenovirus-based GP-rAd5 vaccine [13] depend on CD8 CTL responses [21]. Despite the difficulties associated with defining immune correlates of protection in humans for a disease that rapidly kills such a high fraction of those infected [3], [5] [22], there are indications that survival of symptomatic filovirus disease in a human Sudan virus outbreak was correlated with CTL-mediated immunity [23].

The mosaic vaccine design strategy distills the essential antigenic variation of a collection of sequences by computational recombination of potential CTL epitopes into a small set of chimeric intact protein sequences. The mosaic design algorithm favors common over rare peptide 9-mers and rejects all recombinants that include non-natural peptide 9-mers. The intent of mosaic design is to generate proteins that resemble natural proteins, and are expressed, folded, and processed naturally while maximizing coverage of natural epitope variation [6]. In this way the mosaic proteins can be delivered in vaccines using the same strategies as natural proteins, and should be expressed and processed comparably. Because CTL epitopes are typically 9 amino acids long, 9-mer coverage is optimized, though other epitope lengths are also well covered [14].

So far all HIV mosaic proteins tested have been highly immunogenic, and, as anticipated, elicited responses that are more cross-reactive than responses elicited by natural-strain vaccines [15], [16], [24]. Both CD8 and CD4 T cell responses were enhanced using mosaic vaccine inserts [24]. Although the mosaic proteins were designed to optimize T cell epitope coverage, when used as immunogens they also elicited B cell responses that were comparable to or better than natural strain responses [24], [25]. HIV-1 epitopes from mosaic proteins are processed appropriately – T-cells specific for 13 out of 13 distinct commonly-targeted HIV epitopes isolated from 22 infected individuals were able to recognize human target cells expressing HIV mosaic proteins [26]. The mosaic design approach was subsequently applied to Hepatitis C Virus (HCV) [27], where cocktails of mosaic proteins resulted in improved coverage of potential CTL epitopes from the highly diverse sequence population.

Here we make several comparisons between our mosaic designs and several sets of data. First, we computationally compare coverage of natural epitopes by cocktails of filovirus mosaic proteins with cocktails comprised of an equal number of best-natural sequences. The best-natural sequences are the combination of naturally occurring proteins that provides the best (9-mer) coverage. Second, we compute the coverage of known epitope-containing regions. This gives an indication whether the mosaic strategy retains known good epitopes. Third, we compare our results with the theoretical coverage numbers for a promising ebola GP protein rAd5 vaccine construct [13]. Finally, we show that a mosaic-based ebolavirus vaccine immunogen conferred protective immunity in a mouse model against challenge by a mouse-adapted Zaire ebolavirus strain, protection comparable to protection conferred by a natural Zaire strain vaccine.

## Methods

### Data sets

Our datasets consisted of GP and NP protein sequences because of their established immunogenicity and the comparatively large number of available sequences representing significant viral diversity. Two filovirus sequence datasets were obtained from the Los Alamos Hemorrhagic Fever Virus Database (http://hfv.lanl.gov) and GenBank/GenPept. The first set (“original set”) consisted of 532 filovirus protein and peptide sequences retrieved before the Bundibugyo outbreak. A more recent set of 771 filovirus protein and peptide sequences (the “new set”) formed the basis of a set of designs that included Bundibugyo sequences (the most recent novel outbreak). The original set of input proteins contained 124 GP and 77 NP sequences, the new set 191 GP and 136 NP sequences. We also collected the filovirus epitopes available from the Immune Epitope Database (IEDB) so that we could evaluate the coverage of known CTL epitopes by our mosaic vaccine designs (IEDB sequences are in Dataset S1).

Identical sequences and short sequences identical to part of a longer sequence were removed from both sets of protein sequences so that an unbiased measure of amino acid 9-mer coverage could be calculated. For the original set, 51 GP and 31 NP sequences remained after removal of duplicates. For the new set, 74 GP and 47 NP sequences remained. GenBank accession codes that define the original and new sequence sets are given in the Dataset S2.

The viral amino acid sequences were grouped by protein and by viral genus (*Ebolavirus* and *Marburgvirus*); the Ebola sequences were then categorized into the five species (*Reston*, *Sudan*, *Zaire*, *Bundibugyo*, and *Taï Forest ebolaviruses*). Correct classification of viral sequences was verified by comparing nucleotide phylogenies made using PhyML [28], protein trees generated by RIND [29] and FastTree [30], and by examining sequence annotation. These sequences defined reference sets used for scoring epitope coverage of vaccines, and were also used to prepare input files for the computational design of cocktails of mosaic proteins.

### Computational vaccine coverage evaluation

Coverage of potential epitopes by vaccine candidates is calculated as the mean number of potential CTL epitope 9-mers per sequence in a reference set (either the new set of filovirus proteins, or epitope sequences) that exactly matched a 9-mer in a vaccine. We also calculated the off-by-1 and off-by-2 matches to 9-mers because many such near matches also generate immunological responses. A vaccine in this case is either a cocktail of co-optimized mosaic proteins, a cocktail of best-natural proteins, or a recent vaccine candidate [13].

We tested the robustness of vaccines to the discovery of new viral sequences by designing two groups of mosaic cocktails: mosaics that used the original set of filovirus proteins as input to the mosaic generation and optimization scheme, or mosaics that used the new set of filoviral proteins. The epitope coverage of both sets is reported for the new set of filoviral proteins. This provides an estimate of how much vaccine epitope coverage degrades as new viral sequences are discovered.

We also evaluated the mosaics by computing the number of potentially-rare epitopes present in the cocktail, and by computing the number of non-natural peptides of length 10, 11, or 12 residues present in the mosaic cocktails.

### Mosaic generation

Cocktails of mosaic proteins (i.e. one or more co-optimized mosaic proteins) were generated for each of the 7 groups of filoviral proteins (one group per gene) based on the original set of viral protein sequences. As discussed above, because CTL epitopes are typically 9 amino acids, and because more mosaic proteins are possible when designing for shorter epitopes, we always optimized for 9-mer amino acid coverage. This sacrifices little coverage up to 12-mers, so helper-T-cell epitope coverage is also improved [14]. While this creates the possibility of un-natural *k*-mers of 10 – 12 amino acids spanning recombination breakpoints, this is not a serious problem for these data sets (see Results).

Other than the NP and the L (polymerase) proteins, there are no amino acid 9-mers in common between ebolaviruses and marburgviruses, an unsurprising result in light of the distances in protein phylogenies reported in Figure S1. For proteins with no shared 9-mers in common, the lack of valid recombination sites means that optimization of two mosaic cocktails, one for ebolaviruses and another for marburgviruses, gives 9-mer coverage indistinguishable from a simultaneous optimization of a single cocktail over both viruses. For NP and L proteins, simultaneous optimization of mosaics covering ebolaviruses and marburgviruses into a single cocktail would mix maximally divergent sequences. Given the dearth of structural information on filoviral proteins, this does not seem advisable given our design objective of maintaining “naturalness” of the mosaic proteins. Consequently, we designed separate Marburgvirus and Ebolavirus mosaics.

The number of proteins in each cocktail was increased until coverage approached 100%. Based on the number of wild-type sequences per protein, limits imposed by the physical size of potential vaccine constructs, and because the antigenicity of NP and GP proteins are best characterized, the mosaics optimized against the new set of viral proteins were designed for only NP and GP. All comparisons reported here are for these two proteins.

The strategy to identify mosaic cocktails with good coverage of the various filoviruses began with simultaneous optimization of 9-mer coverage in our new set: NP and GP from ebolaviruses and marburgviruses. This is the same strategy used to optimize HIV-1 mosaic cocktails [6]. As Yusim *et* *al.* [27] previously found for HCV, this strategy is not optimal for under-sampled clades, here most strongly affecting the *Bundibugyo*, *Taï Forest*, and (for some purposes) *Sudan ebolavirus* species. To compensate for under-sampling, we used two alternate strategies. One was a variation of the serial optimization strategy in [27]. The common 9-mers from viruses in the three better-sampled species (*Zaire*, *Sudan*, and *Reston ebolaviruses*) were represented by a single mosaic protein optimized against those species only. This “background” mosaic was included as a fixed sequence in the optimization of a single mosaic protein for *Bundibugyo* and *Taï Forest ebolaviruses*. The background mosaic was then discarded, and a second simultaneous optimization of the full-sized mosaic cocktail was performed with one protein fixed to be the Bundibugyo virus/Taï Forest virus mosaic. The second strategy duplicated sequences from under-sampled species for the design inputs (but not coverage analysis). The use of this strategy for a better-sampled virus would not be desirable because the objective of mosaic protein optimization is to eliminate low-prevalence epitopes in favor of epitopes found in many different viruses. However, in the case of the filoviruses we have little information about what conserved or rare epitopes exist in these species, and duplication of sequences in the input files effectively increases the weight, and hence coverage, of some species in the mosaic optimization process. We report re-weighting as strategy “A” and serial optimization as “B”. In our reported results we simply report the better of these two strategies when not discussing the results of simultaneous non-redundant optimization.

In principle, epitopes that are rare in the population should be excluded from the mosaics because they could conceivably “distract” the CTL response from other cross-protective epitopes. One important feature of designed mosaics is their much lower content of these rare and unique epitopes. However, in contrast to both HIV-1 and HCV, the small number of known filovirus sequences diminishes our ability to identify rare and unique epitopes. In the previous applications of the mosaic method to HIV-1 and HCV, larger numbers of sequences were available: 551 Gag and 1131 Nef sequences for HIV-1, and 176 whole-genomes for HCV. In addition, analysis of HCV mosaics was greatly facilitated by the availability of 3870 sequences over the roughly 100 amino acid long Okamoto region. Thus HIV-1 and HCV mosaics had a reasonable statistical basis to define rare epitopes. Because we used 75 unique GP and 47 unique NP sequences for our optimal design, the concept of rare epitopes is less well-defined for any filovirus species, and impossible to define for the *Bundibugyo* species. “Uniqueness” of potential CTL-epitopes will only be relevant as more sequences become available; until then “unique epitopes” represent a set of *potentially* rare epitopes.

### Virus, mice, and infections

Mouse-adapted ebolavirus has been previously described [31]. Forty female C57BL/6 mice (5–8 weeks) were obtained from the National Cancer Institute (Frederick, MD). Mice were housed under specific-pathogen-free conditions. Research was conducted in compliance with the Animal Welfare Act and other Federal statutes and regulations relating to animals and experiments involving animals and adhered to principles stated in the Guide for the Care and Use of Laboratory Animals [32]. The facility where this research was conducted is fully accredited by the Association for the Assessment and Accreditation of Laboratory Animal Care International. For infection, mice were inoculated intraperitoneally with a target dose of 1000 plaque forming units (30,000×the 50% lethal dose) of mouse-adapted Zaire virus (accession code AF499101) in a biosafety level 4 laboratory. Mice were observed for 28 days after challenge by study personnel and by an impartial third party. Daily observations included evaluation of mice for clinical symptoms such as reduced grooming, ruffled fur, hunched posture, subdued response to stimulation, nasal discharge, and bleeding. Serum was collected from surviving mice and stored at −80°C until plaque assays were completed to confirm virus clearance. Back titration of the challenge dose by plaque assay determined that mouse-adapted Zaire virus infected mice received 950 pfu/mouse.

### Virus-like Replicon Particles and vaccinations

The production of virus-like replicon particles (VRP) expressing Zaire virus or mosaic proteins was performed as previously described [33]. The estimation of replicon titer was performed using previously described methods [34] and flow cytometry-based assay using previously described methods [18]. Groups of 10 mice were injected sub-cutaneously in the dorsal neck region with either 2×10^6^ or 2×10^7^ infectious units of VRPs encoding for either Zaire virus GP (accession code AAD14585) or the most Zaire-like single mosaic GP protein from the 4-protein cocktail optimized against the original sequence set (see Dataset S3). As indicated, some mice received booster vaccination. Mice were bled retro-orbitally 21 days after each vaccination and serum isolated for ELISA.

### Enzyme linked immunosorbance assay

Filovirus specific serum IgG titers were determined by enzyme linked immunosorbance assay (ELISA) using recombinant Zaire virus glycoprotein. Polyvinyl chloride ELISA plates (Dynatech Laboratories, Chantilly, VA) were coated with appropriate antigen diluted in phosphate buffered saline (PBS) over night at 4°C. Plates were blocked with 5% milk protein in PBS/0.02% Tween 20 (Sigma-Aldrich, St. Louis, MO) at room temperature. Serum samples were diluted in blocking buffer supplemented with 1% goat serum and serial 0.5 log dilutions were performed. Coated ELISA plates were incubated with diluted murine serum samples for two hours at room temperature and then washed with PBS/0.02% Tween 20. Plates were incubated with horseradish peroxidase conjugated goat anti-mouse IgG (Rockland, Gilbertsville, PA) diluted in blocking buffer for one hour at room temperature. Plates were washed as described above prior to the addition of 2,2′-azino-bis(3-ethylbenzothiazoline-6-sulphonic acid) (ABTS) substrate (Kirkegaard & Perry Laboratories, Inc, Gaithersburg, MD). Absorbance values were read at 405 nm using a Spectramax plate reader (Molecular Devices, Sunnyvale, CA). To determine cut-off values for each dilution, pre-vaccination serum samples from each individual mouse were run in parallel with post-vaccination and post-challenge samples. Cut-off values for each mouse's serum were calculated by performing replicated dilutions of the pre-vaccination serum. We took the cut-off value to be the average absorbance +3× standard deviation across the replicated dilutions. The end titer for each “post-“ sample is reported as the last dilution to exceed the cut-off value.

## Results

We computationally evaluated three categories of vaccines: cocktails of mosaic proteins (mosaic sequences are given in Dataset S3), collections of “best-natural” proteins (best-natural sequences are given in Dataset S4), and a GP-rAd5 experimental vaccine candidate coding for two proteins, accession codes NP_066246 (Zaire virus GP) and YP_138523 (Sudan virus GP) [13]. Best-natural vaccine means the cocktail of wild-type proteins that gives the best 9-mer coverage of viral proteins for a specified cocktail size. A sequence's epitope coverage is evaluated by calculating mean epitope coverage per sequence of three data sets: the original and new sets of unbiased sequences, and the set of CTL epitope-containing sequences from IEDB. Only comparison with the new sequences and IEDB sequences are reported. The unbiased data sets are unbiased in the sense that they did not contain duplicate sequences (see subsection “Data sets”). Because of its apparent non-pathogenicity to humans, results for Reston virus are not shown, but they are included in the genus-wide coverage scores.

### Overall epitope coverage

**Figure 1:**
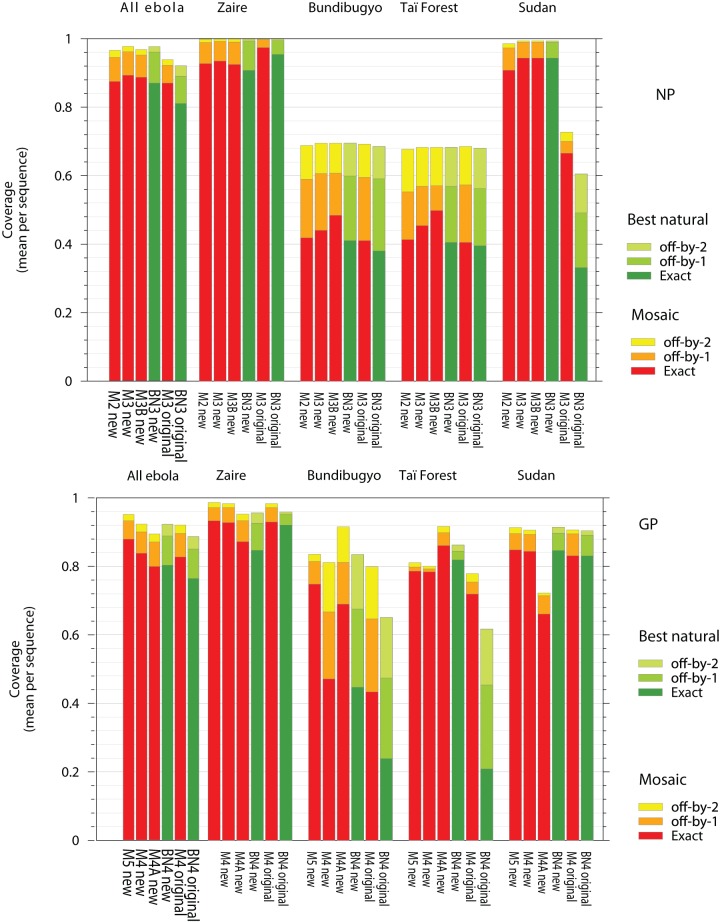
Sequence-averaged 9-mer coverage by vaccines composed of cocktails or proteins. M*n* designates a mosaic cocktail containing *n* co-optimized proteins. BN*n* designates a best natural cocktail containing *n* proteins. “Original” refers to coverage of the new sequence set by a vaccine using the original sequence set as input. “New” refers to coverage of the new sequence set by vaccine designed against the new sequence set. M*n*A refers to a mosaic with a serial optimization strategy. M*n*B refers to re-weighting of clades by the duplication of sequences in the final design set (see Methods).

Figure 1 reports the coverage of epitopes in the new set of ebolavirus sequences by various sizes of mosaic and best-natural NP and GP vaccines. Some of the mosaic vaccines were optimized using the new set of filoviral protein (labeled “new” in Fig. 1) as inputs, and some were optimized using original filovirus proteins (“original”) as inputs. Results for best-natural vaccines are also shown. This allows a direct comparison of epitope coverage between mosaic proteins and the best-natural protein strategies for cocktails consisting of different numbers of proteins. The mosaic cocktails attain exact-match coverage from a few percent to nearly ten percent better than that of the equivalent best-natural vaccine (“M3” vs. “BN3” for NP, “M4” vs. “BN4” for GP), regardless of whether original or new inputs were used to design the mosaic cocktail. The largest improvements of mosaic over best-natural vaccines are observed in cases where there are comparatively few known sequences, for example the original NP vaccine against Sudan virus and original GP vaccine again Bundibugyo and Taï Forest viruses.

As expected, the epitope coverage uniformly increases with the number of proteins included in either a mosaic cocktail or a collection of best-natural proteins (for example compare “M2 new” to “M3 new” for NP). This is true regardless of whether the vaccine was optimized against the new set of filovirus proteins (“new”), or the original set of proteins (“original”). Because of the high diversity of the ebolaviruses, single mosaic proteins provide only 66% and 46% genus-wide exact-match coverage of NP and GP 9-mers respectively (data not shown). The 3-protein NP mosaic cocktail and 4-protein GP mosaic cocktails achieve 89% and 84% exact-match coverage. The diversity of NP and GP is comparable to HIV-1 M-group Gag and Env proteins (Figure S1). This observation suggests a comparison of filovirus mosaics with the HIV-1 M-group mosaics. M-group 2-protein mosaics have been moved into animal vaccine trials. Those 2-protein mosaics provide 62% (Gag) and 38% (Env) exact-match 9-mer coverage and 94% (Gag) and 75% (Env) off-by-two coverage (unpublished data). The filovirus vaccines are comparable to these epitope coverage levels, suggesting that enhanced cross-reactive potential in terms of improved breadth and depth of CTL responses is likely for our ebolavirus vaccines; the advantages are most striking for increasing the cross-reactivity with Taï Forest virus.

### Coverage robustness

**Figure 2:**
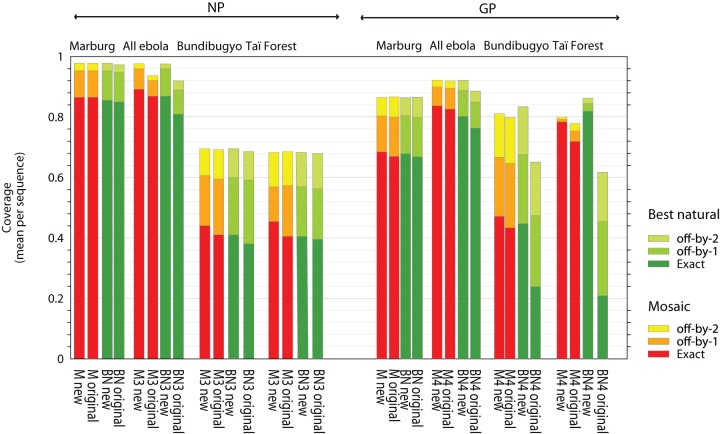
Coverage robustness. The coverage of new protein set viral diversity of nucleoprotein (NP) and glycoprotein (GP) by vaccines designed against original and new protein sequence sets for both mosaic cocktails and best-natural cocktails. Comparisons are shown of *Marburgvirus* and *Ebolavirus* genera, and of *Bundibugyo ebolavirus* and *Tai Forest ebolavirus* species.

An important consideration for a vaccine against idiosyncratic and unpredictably re-emergent viruses like the filoviruses is the stability of vaccine epitope coverage as new species and strains of virus are discovered. To get a sense for how robust the vaccines are to the discovery of new sequences in new outbreaks, we calculated coverage of the new sequence sets by both the old and new vaccine designs (Figure 2). Overall, the performance of the new design NP mosaic cocktail across the variety of ebolaviruses is better than the original mosaic, but for the GP protein this is not always true. Coverage of Zaire virus by the GP mosaics is slightly lower in the new design. This reflects over-optimization of the original design to a single well-sampled species, rather than a defect in the new design. More importantly, the comparisons show that the mosaic design based on the original set of sequences performed much better in every case against the new sequence set than the original best-natural sequences, including GP (red “original” bars are all higher than the corresponding green “original” bars in Fig. 2). It is also important that in every case, the old mosaics are close to the performance of the new best-natural vaccine (“M original” type labels vs. “BN new” type labels).

Mosaic coverage of Bundibugyo virus NP is an interesting case. Even though there were no Bundibugyo virus sequences in the original set, the performance of the original mosaic is within 4% of the new mosaic cocktail (which did include Bundibugyo as part of its design inputs). This suggests that the coverage of the mosaic design is quite stable, even against a new viral species.

### Compensating for viral under-sampling

Coverage by the most optimized mosaics (labels containing “new” in Fig. 1) of sequences belonging to the genus *Ebolavirus* varies significantly between species. Species that have a small number of known sequences tend to be significantly less well-covered by mosaics generated using simultaneous optimization (labels like M2, M3, M4, M5, but not M4A or M3B) than species with relatively large numbers of known sequences. Similarly, coverage by the best-natural strategy (green bars in Fig. 1) varies significantly between species. We used modified mosaic optimization methods (see Methods) to reduce this inter-species variability of epitope coverage.

Applying our new optimization strategies increases the Bundibugyo virus GP coverage for the 4-mosaic design from 47% to 69% (figure legend: M4 to M4A), at the cost of a decline in Sudan virus coverage from 88% to 66%. 5 mosaic proteins (simultaneous optimization) provide ≥85% coverage of the three well-sampled species and ≥75% coverage of Bundibugyo virus and Taï Forest virus. These modified optimization strategies also reduce variability for NP (Fig. 1). Coverage of Bundibugyo virus and Taï Forest virus NP can be increased from 44 and 45%, to 48 and 50%, respectively, using a re-weighting strategy. These increases of 4–5% come at the cost of no more than 1.2% of coverage in the other species.

### Marburgvirus

The limited sequence diversity of the marburgviruses is reflected in the coverage results for NP (88%) and GP (68%) by a single mosaic protein. A two-mosaic cocktail gives excellent coverage of marburgviruses, even for the more variable GP protein. Including off-by-one amino acid matches, the marburgvirus 2-protein NP mosaic cocktail covers more than 98% of 9-mers. 3 and 4 Marburgvirus NP mosaics provide coverage above 99%.

Known marburgvirus diversity is low compared with the ebolaviruses, and the only significantly different new sequences in the new set belong to the genus *Ebolavirus*. The addition of only a few new marburgvirus sequences means that the mean coverage per sequence by the old mosaic cocktail is only about 1.5% lower than the new cocktail for Marburgvirus NP.

### Comparison of mosaic designs with a vaccine candidate

**Figure 3:**
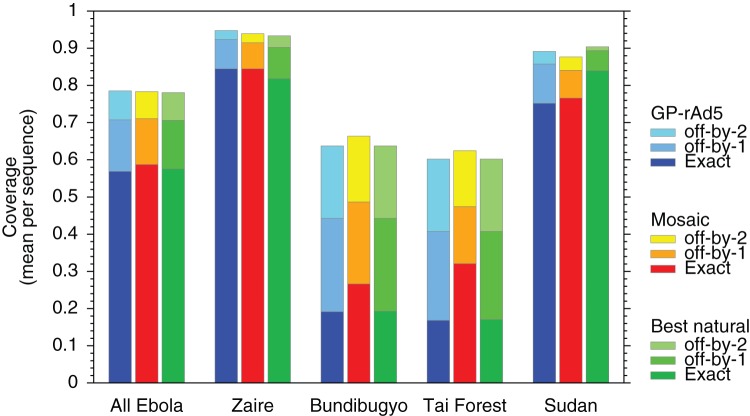
Comparison between vaccine strategies. The computed epitope coverage by the Hensley *et* *al.* GP-rAd5 vaccine proteins, the two 2-protein mosaic cocktail, and the two best-natural proteins. The 2-protein mosaics and best-naturals were optimized against the ebolaviruses excluding Reston virus. The plotted results include Reston virus in the genus-wide coverage results.

We compared our strategy's coverage to the GP-rAd5 vaccine that was tested in macaques by Hensley *et al*. [13]. Because the Hensley vaccine consists of only 2 GP proteins, we compared with a 2-protein GP mosaic cocktail's coverage of the ebolaviruses, and several ebolavirus species separately (Fig. 3). The 2 mosaic cocktail generally shows higher coverage than either GP-rAd5, or the 2-protein best-natural cocktail.

### Unique and absent epitopes and k-mer length

**Figure 4:**
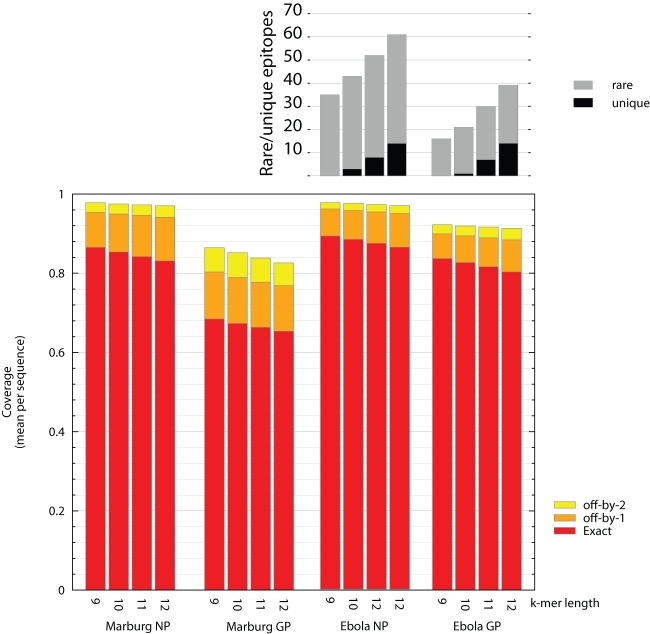
Overall coverage, unique, and absent *k*-mers, as functions of *k*-mer length. Marburgvirus NP and GP data are for a single-protein mosaic cocktail, ebolavirus NP data are shown for a 3 mosaic protein cocktail with simultaneous optimization of all mosaics, Ebolavirus GP data are shown for a 4 mosaic protein cocktail with simultaneous optimization. Ebolavirus unique (grey) and absent (black) *k*-mers. For ebolavirus GP there are 550, 510, 474, and 449 distinct *k*-mers for *k* = 9, 10, 11, and 12; for ebolavirus NP 451, 466, 437, and 371. Marburgvirus shows no unique or absent *k*-mers, for 9≤*k*≤12, and so is not shown in this panel.

The coverage that various vaccine designs provide for the new sequence set for peptide *k*-mer lengths 9–12 is shown in Figure 4. Optimizing for 9–mers means the coverage for longer epitopes is less than optimal, but, as we found for HIV-1 [14], the results in Figure 4 show that this is not a major concern in our designs: our mosaics are optimized to cover 9-mers and *k*-mer coverage typically drops by only 3% between *k* = 9 and 12.

Because the mosaic optimization process only guarantees natural *k*-mers for the design length of epitopes, and we have set *k* = 9, non-natural 10-, 11- and 12-mers may be present in the final mosaics. The number of these non-natural *k*-mers in the mosaic (i.e. that are *absent* from the new set of filoviral proteins) is never more than 3.8% of all *k*-mers (for k = 12), with the absolute number never exceeding 14 (see figure 1a). The number of potentially rare epitopes is invariably larger, rising as high as 60, from a total number that approximately ranges from 400 to 500 depending on *k*-mer length and protein (see the caption for Figure 4 for precise numbers).

### Coverage of known epitopes

There are 25 known CTL epitopes in NP and GP available from the Immune Epitope Database (http://iedb.org/): three for *Marburg Marburgvirus* NP, ten for *Ebolavirus* NP, eight for *Marburg Marburgvirus* GP, and four for *Ebolavirus* GP. Often, epitope-containing sequences are reported rather than exactly the epitope. To allow comparisons with the new sequence set, we computed the fraction of 9-mers covered. We found that the tendency of mosaic design to eliminate low-prevalence 9-mers does not result in disproportionate loss of 9-mers from epitope-containing regions. For marburgvirus GP, mosaic coverage of 9-mers from known epitope-containing regions is superior to the best-natural strategy; for ebolavirus NP the strategies are indistinguishable and for marburgvirus NP mosaics have a slight advantage (data not shown).

### Mosaic *in vivo* immunogenicity and efficacy

**Figure 5:**
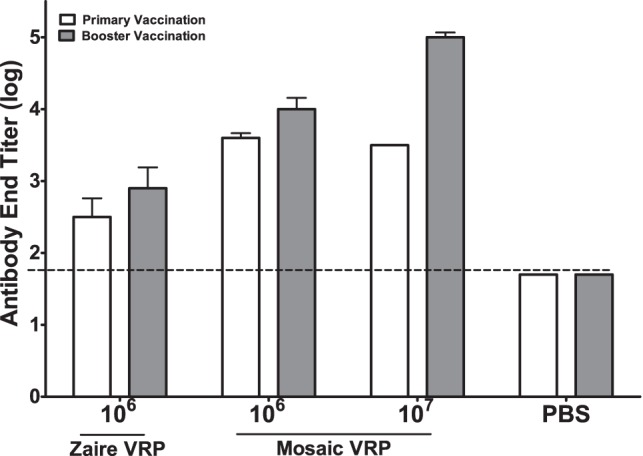
End-point murine IgG antibody titers measured by ELISA. Log antibody (IgG) end titers from female C57BL/6 mice. End titers are against recombinant Zaire virus GP measured by ELISA. The bar graphs show end titers after vaccination with 2·10^6^ plaque-forming units (pfu) of a virus-like replicon particle (VRP) expressing Mayinga strain Zaire virus GP; 2·10^6^ and 2·10^7^ pfu of VRP expressing the a GP mosaic protein; and phosphate-buffered saline (PBS). The mosaic protein is the most Zaire-like from the 4-protein mosaic Ebola virus GP cocktail designed against the original set. White bars show end titer after primary vaccination, grey bars show end titer after a 28-day delayed boosting vaccination. The dotted line is the ELISA cut-off as defined in the text.

**Figure 6:**
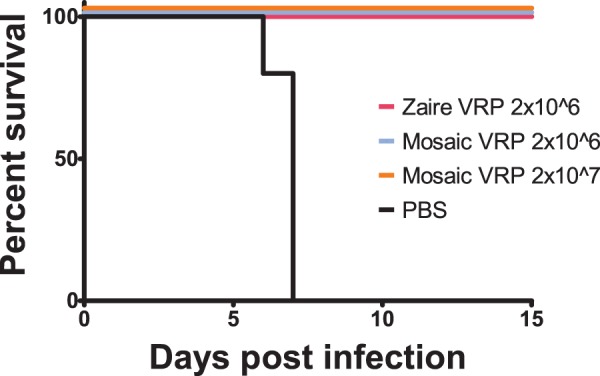
Murine survival curves after Zaire virus challenge. Survival curves for 4 cohorts of 10 female C57BL/6 mice are shown versus time after challenge with 950 plaque-forming units (pfu) of mouse-adapted Zaire virus. One cohort (red line) was vaccinated with 2·10^6^ pfu of a virus-like replicon particle (VRP) expressing GP from Mayinga Zaire virus, the second cohort (blue line) was vaccinated with 2·10^6^ pfu of a VRP expressing a GP mosaic protein, the third cohort (orange) was vaccinated with 2·10^7^ pfu of the same VRP expressing a GP mosaic protein, the fourth cohort served as controls and were sham vaccinated with phosphate buffered saline (PBS). The mosaic protein expressed by the VRP is the most Zaire-like protein from the 4-protein mosaic cocktail optimized against the original set of filovirus sequences. A single mosaic protein from the cocktail was used because experimental resources were limited.

To assess the immunogenicity and efficacy of a mosaic protein, we vaccinated mice with a single mosaic, and compared this to a single natural protein. Because resources were limited for this experiment, we selected for testing the single mosaic protein from a four-protein mosaic cocktail that was the most Zaire virus-like. After vaccination, we assessed the humoral immune response by ELISA to mosaic GP and wild-type GP. Mice vaccinated or vaccinated and boosted with mosaic VRP generated similar IgG antibody responses when compared to the antibody responses of mice receiving the Zaire virus GP VRP vaccine (Fig. 5). The Zaire virus GP VRP vaccine used in these studies is similar to that which has been previously reported as immunogenic [18]. Next, we challenged these mice with mouse-adapted Zaire virus 28 days after receiving their last vaccination. All mice receiving a single vaccination of either mosaic VRP or Zaire VRP survived challenge with the virus (Fig. 6). As expected based on the single vaccination results, all mice that received the booster vaccination also survived the challenge (data not shown). For these initial studies to show immunogenicity of mosaics, we only included the 1×10^6^ pfu dose of Zaire VRP, as that is what has previously been shown to be 100% protective in mice. The natural Zaire vaccine strain had 96.0% coverage of 9 mers in the challenge strain GP (i.e., identical 9-mer matches), while the single mosaic strain had 60.9% coverage, so full protection can be achieved in this model by a mosaic with roughly 60% conservation of potential epitopes. For comparison, the Zaire strain GP on average provides 82.8% of other Zaire strains, and 14.0% coverage of non-Zaire strains, while the single mosaic used in the this study provides 54.7% of other Zaire strains and 23.2% coverage of non-Zaire ebolavirus strains. By increasing the numbers of immunogens in each mosaic cocktail, we can achieve relatively high levels of 9 mer coverage for most Ebola viruses: for example, the 4-protein mosaic pool from which the mosaic discussed here was taken provides 82.2% coverage all of Ebolaviruses (Fig. 1). As noted earlier, a single mosaic protein covers 68% of marburgvirus GP 9-mers, above the level required to achieve protection here using a single ebolavirus mosaic protein.

## Discussion

The filovirus family includes 6 species that cause devastating and often fatal disease in humans and primates. We currently have no clear understanding of the full diversity of the filoviruses, nor has study of the known reservoirs anticipated the emergence of a new strain in a filoviral epidemic or primate epizootic. Because the mosaic design strategy is intended to increase the number of common epitopes and reduce the frequency and number of rare epitopes in a vaccine, mosaic cocktails are highly desirable for these highly variable viruses. With this design, we demonstrate that sufficient mosaic recombination between disparate ebolaviruses is possible for the mosaic method to be applied to them. Calculated epitope coverage levels with 1 to 4 proteins per cocktail are comparable to existing experimental vaccines and other design efforts using the mosaic method (i.e. HIV-1 M-group mosaics).

We also note that a single mosaic vaccine cocktail protective against both marburgvirus and ebolavirus is an unlikely prospect. There are relatively few peptide 9-mers in common between ebolaviruses and marburgviruses. While mosaic recombination is possible between ebolavirus and marburgvirus for their NP and L proteins, the phylogenetic distance between viruses in the two genera raises the question of how “natural-like” such a recombinant would be in terms of expression and processing. Because of the relatively low diversity of polymerase proteins, an L protein mosaic is probably the least unlikely target for a potential pan-filoviral mosaic vaccine. To date, none of the other 5 filoviral protein types share 9-mers between ebolaviruses and marburgviruses. If a truly pan-filoviral mosaic vaccine is developed, it will likely rely on inclusion of separate marburgvirus and ebolavirus antigen pools.

Here we have discussed making NP and GP mosaic cocktails. While we have not discussed the viral polymerase (L protein), we know that mosaic recombination of L is possible. We have omitted such results here because of the relatively small size of NP and GP make them more amenable to inclusion in a cocktail of proteins in a vaccine construct. Additionally, relatively few vaccines trials have targeted L. The L protein has a significant number of known sequences and if it were desirable for reasons of antigenicity, mosaics of L protein could be constructed. The other four viral proteins are unlikely targets for mosaic vaccine design at this time due to the relatively small number of known sequences.

We used several alternate optimization strategies to reduce the variability of coverage between species. Our use of serial rather than simultaneous optimization was found, as in the case of HCV vaccine design, to reduce inter-clade variation in coverage, without sacrificing much overall coverage. We introduced here the re-weighting of clades by the simple expedient of duplication of sequences from under-represented clades, also to good effect.

Significant cross-protection was experimentally demonstrated between two viral species by Hensley *et* *al.* [13]. Here we have shown by computational means that the mosaic strategy can achieve coverage of potential CTL epitopes (amino acid 9-mers) superior to that computed for Hensley's GP-rAd5 construct, while retaining most experimentally-determined CTL epitope-containing regions. Furthermore, our design shows smaller inter-species variation and better coverage than can be achieved with an equal number of the best-naturally occurring proteins. The mosaic cocktails also exhibit better coverage robustness upon discovery of new strains (including Bundibugyo virus) than best-natural cocktails. This may be of great importance in the future as new filoviruses are discovered.

Because general prediction of vaccine protein processing and presentation to CTLs is not currently possible, it is especially important to avoid introducing potential epitopes that are absent from the design target. While the mosaic design process guarantees this result for peptide 9-mers, it cannot make this guarantee for 10 – 12 *k*-mers. It is a promising result of this study that the mosaic design process is introducing 4 times fewer potential epitopes *absent* from the new sequence set than it retains from the set of potentially-*rare* epitopes (Figure 4). While we would also like to eliminate epitopes that are *rare* in the design target from the mosaic cocktails because of their potential to distract the CTLs from cross-protective epitopes, the small number of available filovirus sequences do not provide a good statistical basis for defining rare epitopes.

Our demonstration of IgG antigenicity by ELISA and of heterologous protection in mice using a mosaic protein vaccine establishes that the chimeric mosaic protein discussed here can be expressed, processed and presented for immunological surveillance, and that protective responses can be evoked. This critical experiment should be regarded as a proof-of-concept for the feasibility of synthetic mosaic immunogens for ebolavirus GP. Based on our previous work with other viruses, we anticipate that a full mosaic cocktail could induce broad immune responses, and possibly broadly protective responses, but this remains to be tested.

A pan-Ebola virus vaccine seems possible in the near future based on experiments and our computational work. A pan-filoviral vaccine would be a singular advance in the management of the filoviruses. A successful vaccine would find uses in the control of human outbreaks, the preservation of non-human primate species in Africa and a concomitant reduction in transmission to people, the protection of laboratory workers and clinical teams, and a disincentive to the use of filoviruses as bioterrorism or bio warfare agents.

Our vaccine design effort provides support for the conclusion that explicit consideration of viral diversity and how best to cover it improves the process of vaccine development. This observation is especially important for highly-variable pathogens. The mosaic proteins' superior coverage robustness after the discovery of new species or strains also makes mosaic cocktails a better candidate for new vaccines, particularly when the viral reservoir is only partly characterized. This consideration does not appear to be a current design objective of many vaccine design efforts, but is a worthy goal for the highly variable viruses.

## Supporting Information

Figure S1 **Comparative Protein Phylogeny.** Unrooted maximum-likelihood protein phylogenies for Gag and Env protein (HIV-1 M-group) and NP and GP (filoviruses). HIV-1 clades and filoviral speices are indicated by the labels. All trees are drawn to the same scale to facilitate comparison of the phylogenetic diversity of HIV-1 and the filoviruses. All trees were made using the same tree-building methodology. All alignments used to make these trees were gap-stripped. HIV-1 M-group alignments are the reference alignments from the Los Alamos HIV Database (hiv.lanl.gov). Filovirus alignments were made using muscle to align the new filovirus protein set. Protein maximum-likelihood phylogenies were made using FastTree with default settings.
(PDF)

Dataset S1 **Epitope-containing sequences from the IEDB.** All epitope containing sequences from the Immune Epitope Database used to determine known epitope coverage are reported in this supplementary information.
(ZIP)

Dataset S2 **Catalog of sequence accession codes.** The two tables and explanatory text give the accession codes of all sequences in the new and old sequence sets.
(PDF)

Dataset S3 **Mosaic protein cocktail sequences.** Amino acid sequences for all proteins in the mosaic cocktails are given in the archive file. Each set of sequences for a cocktail is given in a single file.
(ZIP)

Dataset S4 **Best-natural protein cocktail sequences.** Amino acid sequences for all proteins in the various best-natural cocktails are given in the archive file. Each set of sequences for a cocktail is given in a single file.
(ZIP)
