# Supplementary figures and images for: Designing and Testing Broadly-Protective Filoviral Vaccines Optimized for Cytotoxic T-Lymphocyte Epitope Coverage
Source: PLoS One. 2012 Oct 3;7(10):e44769. doi: 10.1371/journal.pone.0044769 (PMC3463593; doi:10.1371/journal.pone.0044769)

**HIV-1 M-group GAG**

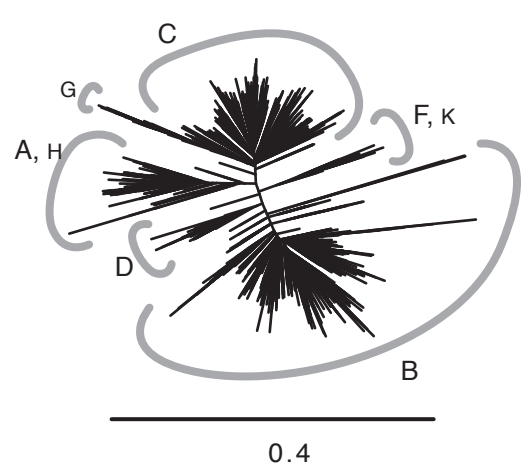

**HIV-1 M-group Env**

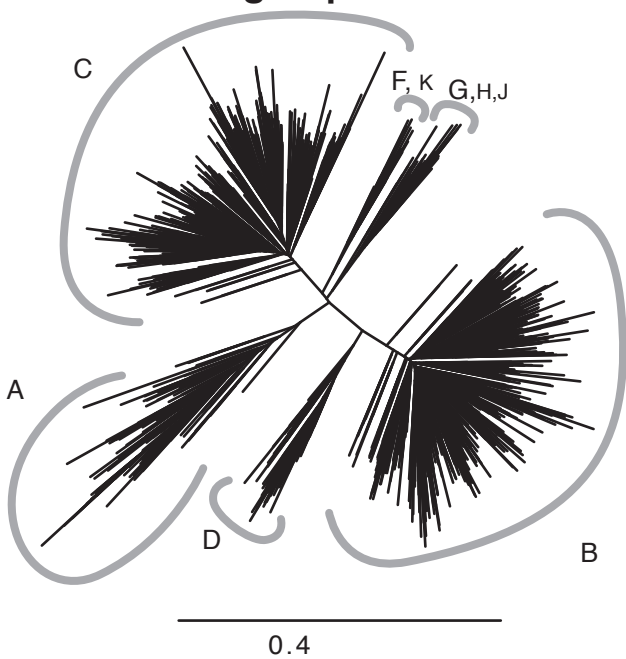

**filovirus NP**

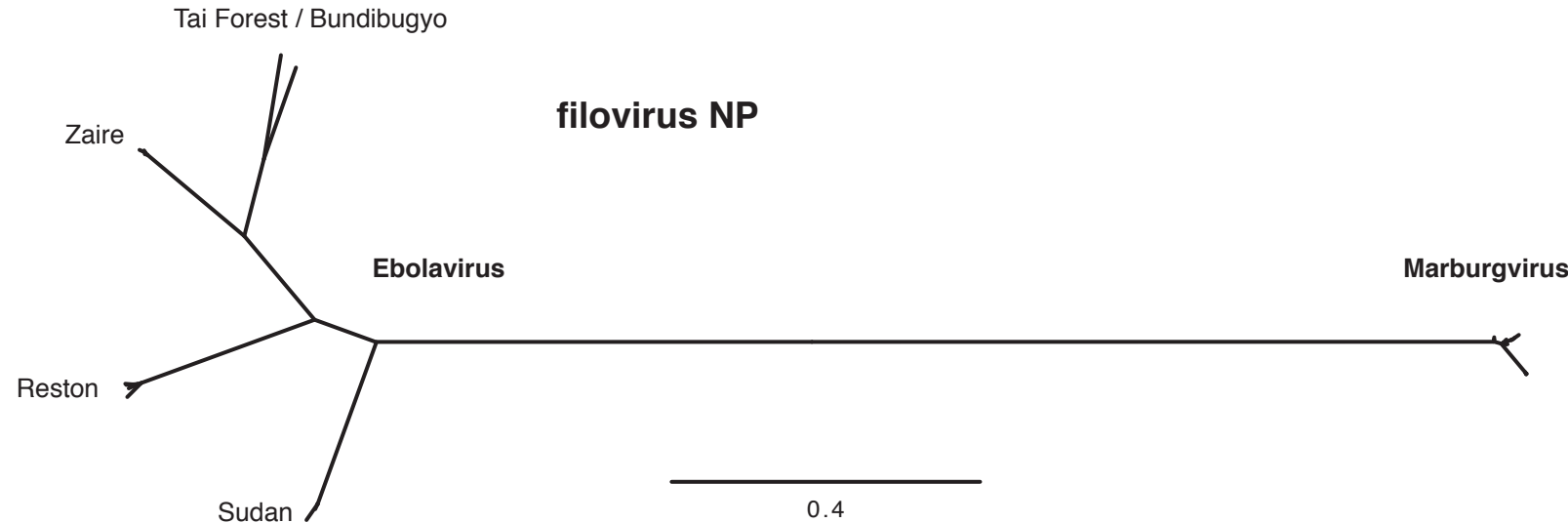

**filovirus GP**

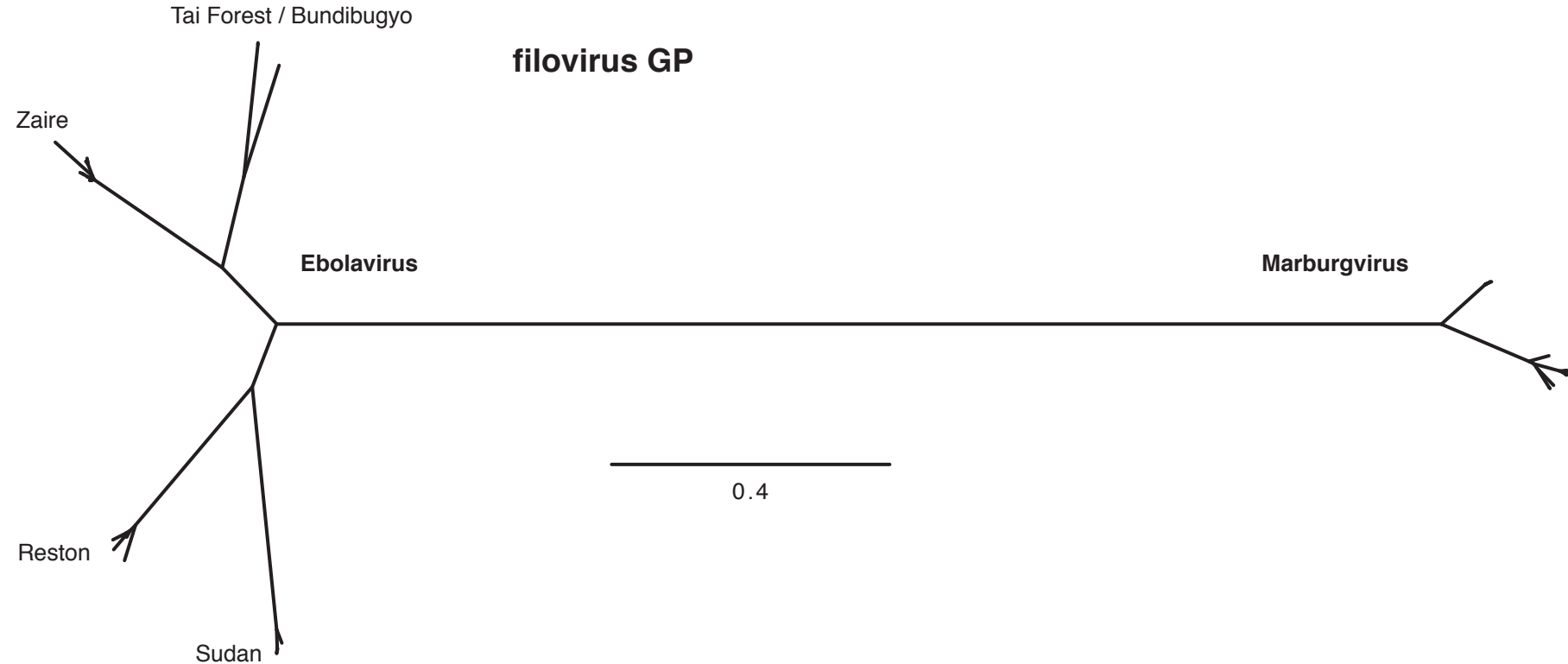

Supplement: Figure S1 — Comparative Protein Phylogeny. Unrooted maximum-likelihood protein phylogenies for Gag and Env protein (HIV-1 M-group) and NP and GP (filoviruses). HIV-1 clades and filoviral speices are indicated by the labels. All trees are drawn to the same scale to facilitate comparison of the phylogenetic diversity of HIV-1 and the filoviruses. All trees were made using the same tree-building methodology. All alignments used to make these trees were gap-stripped. HIV-1 M-group alignments are the reference alignments from the Los Alamos HIV Database (hiv.lanl.gov). Filovirus alignments were made using muscle to align the new filovirus protein set. Protein maximum-likelihood phylogenies were made using FastTree with default settings. (PDF) [file pone.0044769.s001.pdf]
