# Supplementary material for: Designing and Testing Broadly-Protective Filoviral Vaccines Optimized for Cytotoxic T-Lymphocyte Epitope Coverage
Source: PLoS One. 2012 Oct 3;7(10):e44769. doi: 10.1371/journal.pone.0044769 (PMC3463593; doi:10.1371/journal.pone.0044769)
Supplement: Dataset S2 — Catalog of sequence accession codes. The two tables and explanatory text give the accession codes of all sequences in the new and old sequence sets. (PDF) [file pone.0044769.s003.pdf]

S3: supporting information: catalog of sequence accession codes

The two tables in this supporting information section give the accession codes of all sequences used as inputs to the pan-filoviral design reported in this paper. The sets of all Ebolavirus and Marburgvirus protein and peptide sequences available through the U. S. National Center for Bioinformatics at the time of design were larger than the lists in the tables here because some sequences contained redundant information. As explained in the main body of the text, sequences that where either identical to another sequence, or are a shortened version of a longer sequence, were excluded. This allowed the elimination of all replicate information when necessary or desirable, and allowed the controlled duplication of sequences in a phylogenetically meaningful way when necessary to control for under-sampling of some viral species. Non-redundant sets of proteins (i. e. precisely the sets of proteins refered to in each column of the tables here) were used to calculate the coverage of peptide 9-, 10-, 11-, and 12-mers (i. e. potential epitopes) and to make the baseline simultaneously optimized mosaic cocktail designs discussed in the main text. Other designs that attempted to control for under-sampling of viral species duplicated sequences within poorly-samples species.

The two proteins reported here, nucleoprotein (NP) and glycoprotein (GP) are listed in each table; "new" and "old" column headings refer to the inputs for the primary vaccine candidates ("new") and the inputs used to design mosaics for testing the erosion of epitope coverage with time ("old").

Marburgvirus Accession Codes			
NP		GP	
new	old	new	old
AAR85453	AAA46563	AAC40458	AAC40458
ABE27040	AAQ55255	AAR85463	AAQ55258
ABE27075	AAR85453	ABE27078	AAR85456
ABE27082	ABE27012	ABE27085	ABE27015
ABE27089	ABE27040	ABE27092	ABE27071
ABS17548	ABE27068	ABF46667	ABE27078
ACD13002	ABE27075	ABF46670	ABE27085
ACD13016	ABE27082	ABS17551	ABE27092
ACT79212	ABE27089	ACD13018	ABF46667
ACT79219	CAA78114	ACT79215	ABF46670
ACT79226	CAA82536	ACT79222	P35254
ACT79240	YP_001531153	ACT79229	YP_001531156
CAA78114		ACT79236	
P27588		ACT79243	
P35263		P35253	
Q1PD53		P35254	
Q6UY69		Q1PD50	
YP_001531153		Q1PDC7	
		Q6UY66	

Table 1: Marburgvirus accession codes for protein sequences used to design pan-filoviral vaccine candidates.

Ebolavirus Accession Codes			
NP		GP	
new	old	new	old
AAA42977	2102210A	AAA96744	AAA96744
AAG40164	AAG40164	AAL25818	AAC54884
AAL25815	AAL25815	AAM76034	AAL25818
AAM76031	AAM76031	AAM76035	AAM76034
AAV48574	AAQ55045	AAN37507	AAM76035
ABW34747	AAV48574	AAQ55048	AAN04451
ABW34748	ABW34747	AAQ55049	AAN37507
ABW34749	ABW34748	AAQ55050	AAQ55048
ABW34750	ABW34749	ABW34738	AAQ55049
ABW34752	ABW34750	ABW34739	AAQ55050
ABW34756	ABW34751	ABW34740	ABW34738
ABW34757	ABW34752	ABW34741	ABW34739
ABW34758	ABW34755	ABW34742	ABW34740
ABX75364	ABW34756	ABW34743	ABW34741
ABY75321	BAB69003	ABW34744	ABW34742
ACI28620	CAA70541	ABW34746	ABW34743
ACI28629	NP_066243	ABX75368	ABW34744
ACR33187	NP_690580	ABX75369	ABW34746
ACT22784	Q9QP77	ABY75324	NP_066248
ACT22792	YP_138520	ABY75325	NP_690583
ACT22807		ACI28623	O11457
O72142		ACI28624	O11458
P18272		ACI28628	P60170
Q5XX08		ACI28632	P60172
Q8JPY1		ACI28633	P87666
Q91DE1		ACI28637	P87670
Q9QCE9		ACR33190	P87671
Q9QP77		ACR33191	Q05320
		ACT22787	Q66798
		ACT22788	Q66799
		ACT22794	Q66810
		ACT22795	Q66811
		ACT22802	Q66814
		ACT22803	Q7T9E0
		NP_690583	Q89569
		NP_690584	Q89853
		O11457	Q91DD7
		O11458	Q91DD8
		P60173	YP_138523
		P87666	
		P87670	
		P87671	
		Q05320	
		Q66798	
		Q66799	
		Q66800	
		Q66810	
		Q66811	
		Q66814	
		Q7T9D9	
		Q7T9E0	
		Q89569	
		Q89853	
		Q91DD7	
		Q91DD8	

Table 2: Ebolavirus accession codes for protein sequences used to design pan-filoviral vaccine candidates.
